# Supplementary material for: Functional genetic encoding of sulfotyrosine in mammalian cells
Source: Nat Commun. 2020 Sep 24;11:4820. doi: 10.1038/s41467-020-18629-9 (PMC7515910; doi:10.1038/s41467-020-18629-9)
Supplement: Supplementary file 1 — Supplementary Information [file 41467_2020_18629_MOESM1_ESM.pdf]

SUPPLEMENTARY INFORMATION

Functional genetic encoding of sulfotyrosine in mammalian cells

Xinyuan He[1], Yan Chen[2#], Daisy Guiza Beltran[3#], Maia Kelly[2#], Bin Ma[2], Justin Lawrie[2], Feng Wang[4], Eric Dodds[2], Limei Zhang[3], Jiantao Guo[2*], Wei Niu[1*]

1. Department of Chemical & Biomolecular Engineering, University of Nebraska-Lincoln, Lincoln, Nebraska, 68588, United States.

2. Department of Chemistry, University of Nebraska-Lincoln, Lincoln, Nebraska, 68588, United States.

3. Department of Biochemistry, University of Nebraska-Lincoln, Lincoln, Nebraska, 68588, United States.

4. Institute of Biophysics, Chinese Academy of Sciences, Beijing, China

# These authors contributed equally to this work.

*To whom correspondence should be addressed: wniu2@unl.edu, jguo4@unl.edu

Supplementary Information

Table of Contents

Table 1. Primer list. Page 3

Table 2. Strain and plasmid list. Page 5

Table 3. X-ray data collection and refinement statistics. Page 7

Supplementary Fig. 1 Selection of sulfotyrosine tRNA synthetase (sTyrRS) Page 8

Supplementary Fig. 2. Flow cytometry analyses. Page 9-10

Supplementary Fig. 3 Confocal images of 293T cells expressing the evolved sTyrRS and an EGFP mutant that contained two amber mutations at position Tyr40 and Asn150. Page 11

Supplementary Fig. 4 Incorporation of sulfotyrosine into EGFP in mammalian cells using evolved sTyrRS. Page 12

Supplementary Fig. 5 Mass spectrometry analysis of sulfotyrosine incorporation intro protein in mammalian cells. Page 13

Supplementary Fig. 6 Structural analysis of sTyrRS. Page 14

Supplementary Fig. 7 Site-specific incorporation of sulfotyrosine into CXCR4. Page 15

Supplementary Fig. 8 Active site close-ups. Page 17

Supplementary Fig. 9 Original SDS-PAGE of Supplementary Fig. 4b Page 18

Supplementary Fig. 10 Original blot of Fig. 4a and Supplementary Fig. 7c Page 19

Supplementary Fig. 11 Original SDS-PAGE of Supplementary Fig. 7c and 7d Page 20

Supplementary Fig. 12 Original blots of Fig. 4b Page 21

References Page 22

Table 1. Primer list.

Primers	Sequences (5'->3')	Usage
EcTyrRS-Y37-F	tgcggcttcgatcctaccgc	*Ec*TyrRS library
EcTyrRS-Y37-R	gcggtaggatcgaagccgcaMNNgagcgcgatcgggccttg	*Ec*TyrRS library
EcTyrRS-L71-F	gtaggcggcgcgacgggtct	*Ec*TyrRS library
EcTyrRS-L71-R	agacccgtcgcgccgcctacMNNcgcaaccggcttgtggcc	*Ec*TyrRS library
EcTyrRS-W129-F	tttggcaatatgaatgtgctg	*Ec*TyrRS library
EcTyrRS-W129-R	gtcagcacattcatattgccaaaMNNgtcatagttgttcgccgcga	*Ec*TyrRS library
EcTyrRS-D182-F	ttcgcctgtctgaacaaaca	*Ec*TyrRS library
EcTyrRS-D182-R	tgtttgttcagacaggcgaaMNNataaccctgcaacaggttgt	*Ec*TyrRS library
EcTyrRS-lib-F	ccctttcttccttgtttcttttt	*Ec*TyrRS library
EcTyrRS-lib-R	taaacgtcggcatccgcagtgtt	*Ec*TyrRS library
PTDH3-1	agcacagatgcttcgttgcttgcatgctagcgttgaatgttagcgtcaacaac	pTDH3 amplification
PTDH3-2	attcttcacctttaaccattttgtttgtttatgtgtgtttattcg	pTDH3 amplification
PTDH3-3	aaacaaaatggttaaaggtgaagaattattcactgg	yeGFP amplification
yeGFP-N149TAG-F	aactctcactaggtttacatcatggctgacaaac	N149TAG mutation
yeGFP-N149TAG-R	atgtaaacctagtgagagttatagttgtattcc	N149TAG mutation
yeGFP-8xHis-R	tcaatggtgatggtgatgatgatggtgaccggttttgtacaattcatccatacc	yeGFP amplification
pcDNA-sTyrRS-F	tcccaggtccaactgcacggaagcttgccaccatggcaagcagtaacttga tt	sTyrRS amplification
pcDNA-sTyrRS-R	gaggctgatcagcgggtttaaacgggcccttatttccagcaaatcagacagt	sTyrRS amplification
eGFP 150TAG-F	acaacagccactaggtctatatcatggccgacaag	N150TAG mutation
eGFP 150TAG-R	catgatatagacctagtggctgttgtagttgtact	N150TAG mutation
CXCR4-F	cccaagcttgccaccatggaggggatcagtatat	CXCR4 amplification
CXCR4-R	aacgggcccttagctggagtgaaa	CXCR4 amplification
21TAG-F	accgaggaaatgggctcaggggactaggactcc	Y21TAG mutation
7F12F-R	gagcccatttcctcggtaaagttatctgaagtaaatatactg	Y7F, Y12F mutation
CXCR4-EGFP-1	acaggtgtccactcccaggt	CXCR4-P38 amplification
CXCR4-P38-R	tgctcacggtggcgaccggtggatcacctttattgaaattagcattttc	CXCR4-P38 amplification
CXCR4-EGFP-3	ggtgatccaccggtcgccaccgtgagcaagggcgaggagc	EGFP amplification
CXCR4-EGFP-4	cgggtttaaacgggcccttagtggtggtggtggtggtgctcgagcttgtacag ctcgtcc	EGFP amplification
pET30b-sTyrRS-F	taactttaagaaggagatatacatatggcaagcagtaacttgatt	sTyrRS amplification
pET30b-sTyrRS-R	agtggtggtggtggtggtgctcgagtttccagcaaatcagacagt	sTyrRS amplification

T7-Tyr-F	gaaattaatacgactcactatagggtggggttcccgagcggc	T7-*Ec*Tyrosyl tRNA amplification
T7-Tyr-R	tggtggtgggggaaggatt	T7-*Ec*Tyrosyl tRNA amplification
SUMO-sTyrRS-F	ctcacagagaacagattggtggatctggaggatccgcaagcagtaacttg attaa	sTyrRS amplification
SUMO-sTyrRS-R	cagcggtttctttaccagactcgagttattaacgttttgccgcctgtaaac	sTyrRS amplification
gRNA-F	caccgcacttcagataactacaccg	CXCR4 sgRNA
gRNA-R	aaaccggtgtagttatctgaagtgc	CXCR4 sgRNA
T7E1-F	aatgtagtaaggcagccaacaggcg	T7E1 assay amplification
T7E1-R	agttaatcactgcccctcctagcag	T7E1 assay amplification

Table 2. Plasmid list.

Plasmid	characteristics	source
p*Ec*TyrRS-lib	Amp^R, TRP1, pADH-EcTyrRS, $tRNA_{CUA}$	Ref 1
pGADGAL4	Amp^R, LEU2, pADH1-GAL4	Ref 1
pDZ276	Amp^R, URA3, pMET17-2xyeGFP	Addgene 35194 Ref 2*
pyeGFP-N149TAG	Amp^R, LEU2, pTDH3-yeGFP N149TAG-8xHis	this study
pcDNA3.1-AzFRS	Amp^R, CMV-AzFRS, U6-$BstRNA_{CUA}$	Ref 3
pEGFP	Amp^R, 3x$BstRNA_{CUA}$, CMV-EGFP Y40TAG	Ref 4
pEGFP-double	Amp^R, 3x$BstRNA_{CUA}$, CMV-EGFP Y40TAGN150TAG	this study
pEcTyrRS	Amp^R, CMV-*Ec*TyrRS, U6-$BstRNA_{CUA}$	this study
psTyrRS	Amp^R, CMV-sTyrRS, U6-$BstRNA_{CUA}$	this study
pCEP4-FLAG-CXCR4	Amp^R, CXCR4	Addgene 98947 Ref 5*
pCXCR4	Amp^R, CMV-CXCR4	this study
pCXCR4(FFF)	Amp^R, CMV-CXCR4 Y7F, Y12F, Y21F	this study
pCXCR4(FF 21TAG)	Amp^R, CMV-CXCR4 Y7F, Y12F, Y21TAG, U6-$BstRNA_{CUA}$	this study
pCXCR4-EGFP	Amp^R, CMV-CXCR4-EGFP	this study
pCXCR4(21TAG)-EGFP	Amp^R, CMV-CXCR4-EGFP Y21TAG U6-$BstRNA_{CUA}$	this study
pCXCR4(N38)-EGFP	Amp^R, CMV-CXCR4(N38)-EGFP	this study
pCXCR4(N38-FFF)-EGFP	Amp^R, CMV-CXCR4(N38)-EGFP Y7F, Y12F, Y21F	this study
pCXCR4(N38-FF 21TAG)-EGFP	Amp^R, CMV-CXCR4(N38)-EGFP Y7F, Y12F, Y21TAG, U6-$BstRNA_{CUA}$	this study
pET30b	Kan^R, protein expression vector in *E. coli*	Novagen
pET30b-sTyrRS-c1	Kan^R, P_{T7}-sTyrRS-c1	this study
pET30b-sTyrRS-c2	Kan^R, P_{T7}-sTyrRS-c2	this study
pET-6xHis-SUMO	Amp^R, protein expression vector in *E. coli* (DE3)	Zhang lab
pET-6xHis-SUMO-sTyrRS	Amp^R, P_{T7}-sTyrRS	this study

pSpCas9(BB)-2A-GFP (PX458)	Amp^R, CMV-SpCas9-2A-GFP	Addgene 48138<br>Ref 6*
pSpCas9(BB)-2A-GFP-sgRNA(CXCR4)	Amp^R, CMV-SpCas9-2A-GFP, U6-sgRNA (CXCR4)	this study

* Plasmid pDZ276 (pURA MET28 PCP-2x-yeGFP) was a gift from Robert Singer (Addgene plasmid # 35194 ; http://n2t.net/addgene:35194 ; RRID:Addgene_35194). Plasmid pCEP4-FLAG-CXCR4 was a gift from Erik Procko (Addgene plasmid # 98947 ; http://n2t.net/addgene:98947 ; RRID:Addgene_98947). Plasmid pSpCas9(BB)-2A-GFP (PX458) was a gift from Feng Zhang (Addgene plasmid # 48138 ; http://n2t.net/addgene:48138 ; RRID:Addgene_48138)

Table 3. Data collection and refinement statistics.

**Data collection**[a]	
Space group	$P2_1$
Cell dimensions	
a, *b*, *c* (Å)	38.1, 93.9, 99.9
α, β, γ (°)	90.0, 92.6, 90.0
Wavelength (Å)	0.97928
Resolution (Å)	38.05–1.78 (1.88–1.78)
No. reflections	67523
Rsym	0.030 (0.753)
$I / \sigma I$	14.1 (1.1)
Completeness (%)	98.4 (97.2)
Redundancy	6.7 (6.5)
$CC_{1/2}$(%)	99.9 (61.2)
**Refinement**	
Resolution (Å)	38.05–1.78 (1.88–1.78)
R_{work} / R_{free}	0.182/0.223
Protein residues	5–322
Ligands	2
Solvent molecules	404
Average B-factors (Å^2)	
Protein and ligands	37.2
Solvent	46.9
R.m.s deviations	
Bond lengths (Å)	0.007
Bond angles (°)	1.40
Ramachandran statistics	
Favored regions (%)	95.9
Allowed regions (%)	4.1
Outliers (%)	0
PDB ID	6WN2

[a]The highest resolution shell statistics are shown in parentheses.

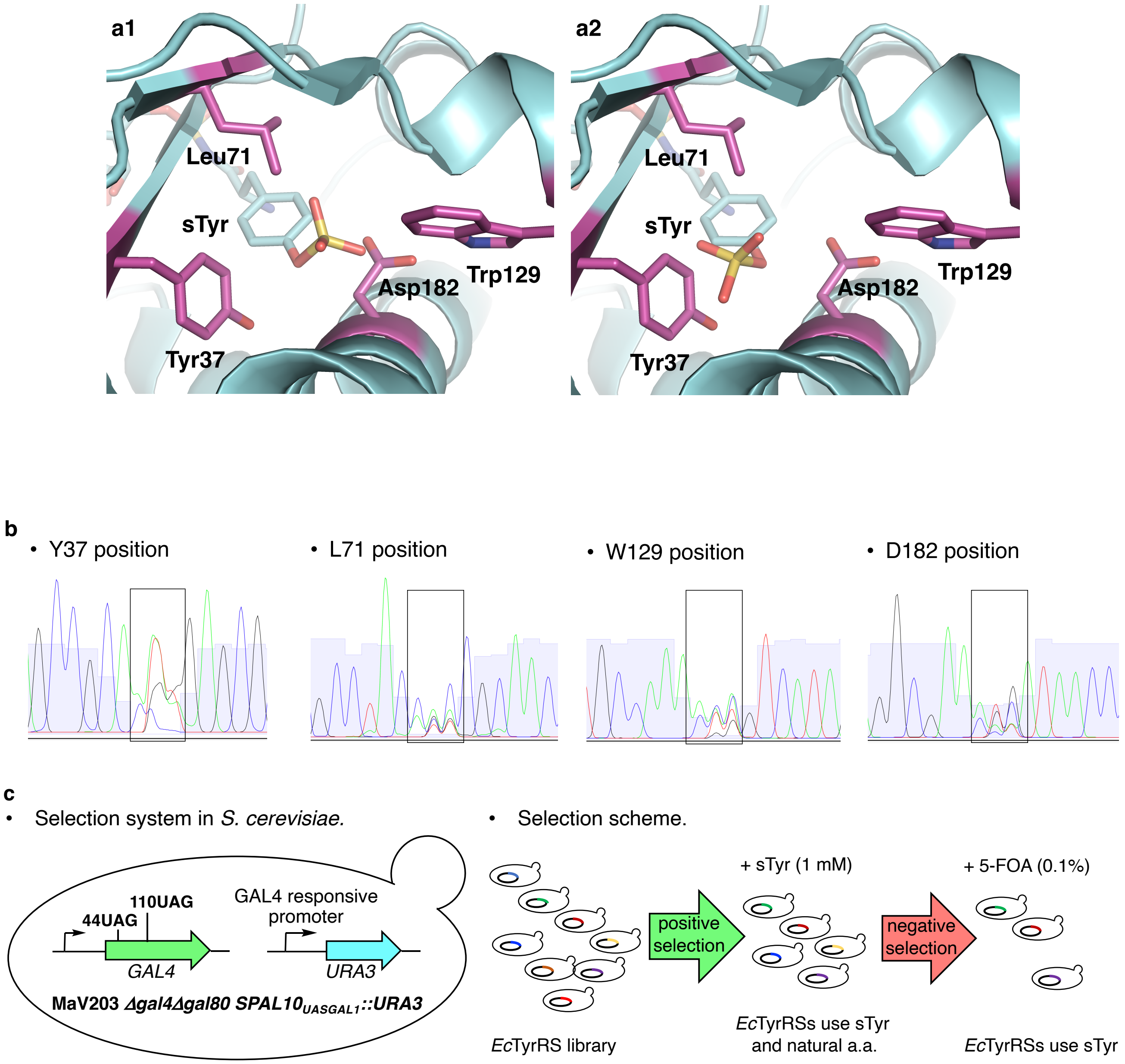

Fig. 1 Selection of sulfotyrosine tRNA synthetase (sTyrRS) candidates. **a** Identification of key residues for the *Ec*TyrRS library. The model was built using the structure of *E. coli* wild-type tyrosyl-tRNA synthetase (PDB ID: 1X8X). Protein ribbon diagrams are colored cyan. Side chains of the four library residues are shown as sticks in purple. Carbons of sulfotyrosine ligand are in cyan, oxygens in red, and sulfur in yellow. Supplementary Fig. a1 and Fig. a2 show two conformations of sTyr by rotation of the C-O single bond. **b** DNA sequencing results of the *Ec*TyrRS mutant library. **c** Selection of *Ec*TyrRS library in *S. cerevisiae*.

a

sTyrRS, no sTyr

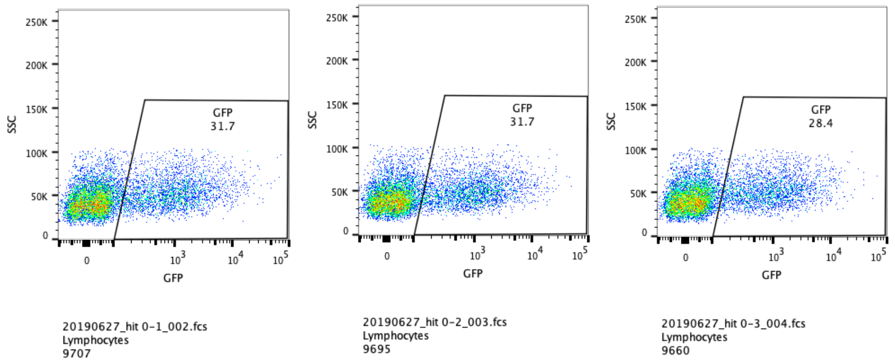

sTyrRS, 1 mM sTyr

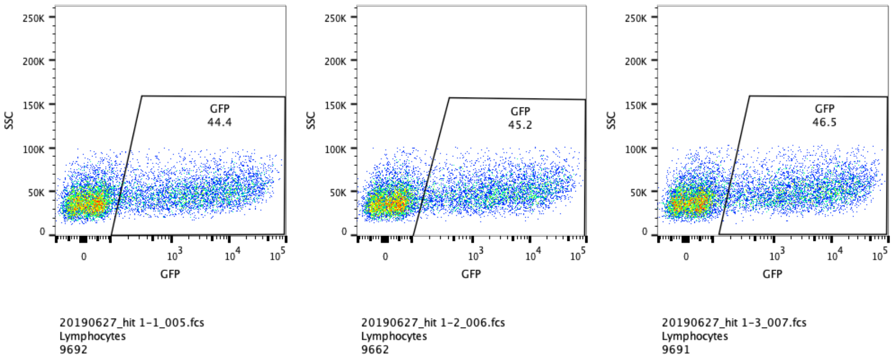

AzFRS, no AzF

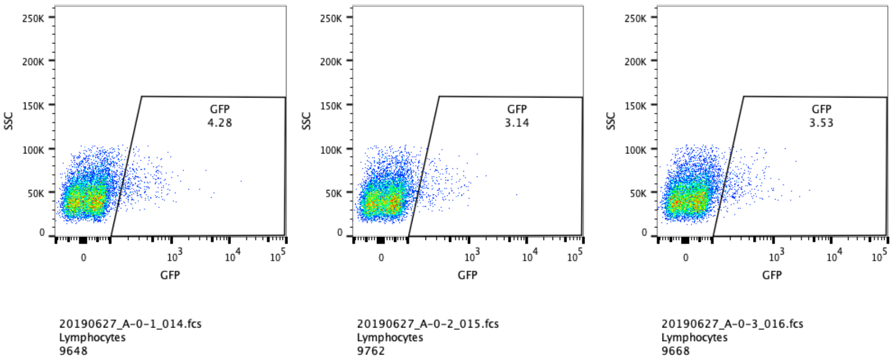

AzFRS, 1 mM AzF

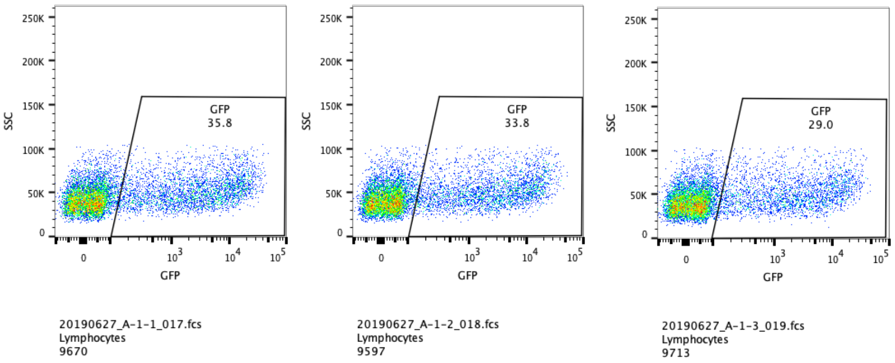

wt *Ec*TyrRS

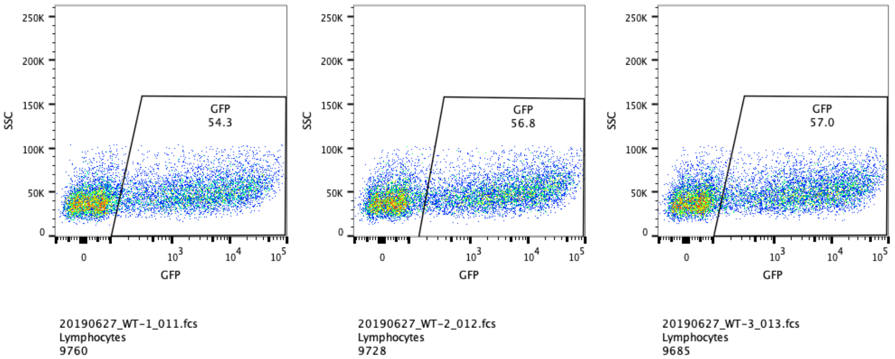

b

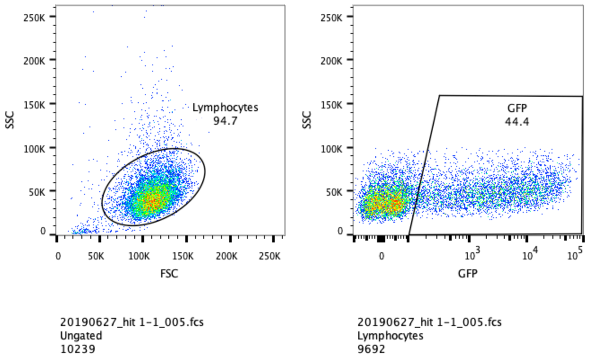

Fig. 2 Flow cytometry analysis. a Flow cytometry analyses of 293T cells expressing the evolved sTyrRS, AzFRS, and wild-type *Ec*TyrRS, each with an EGFP mutant that contains an amber mutation at position Tyr40. **b** Gating strategy for flow cytometry analysis.

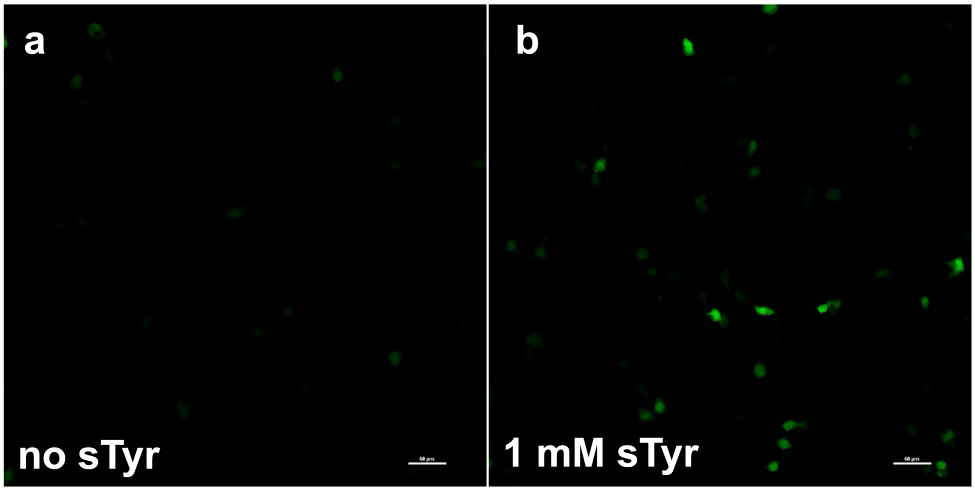

Fig. 3 Confocal images of 293T cells expressing the evolved sTyrRS and an EGFP mutant that contained two amber mutations at position Tyr40 and Asn150. **a** In the absence of sTyr. **b** In the presence of 1 mM sTyr. Scale bars, 50 μm.

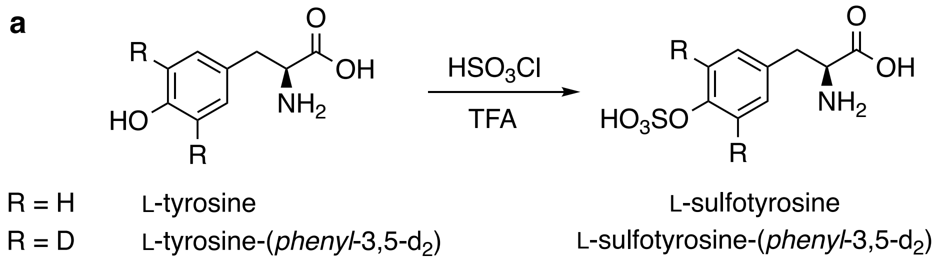

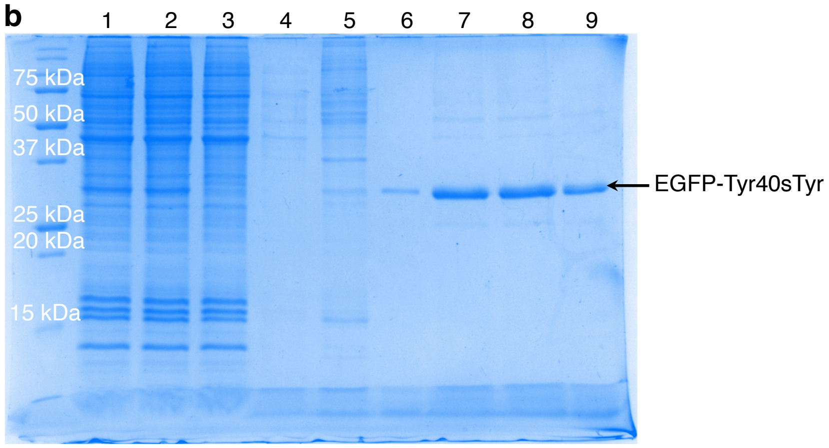

Fig. 4 Incorporation of sulfotyrosine into EGFP in mammalian cells using evolved sTyrRS. a Chemical synthesis of deuterium labelled L-sulfotyrosine-(*phenyl*-3,5-d_2). **b** SDS-PAGE analysis of EGFP-Tyr40sTyr purification from 293T cells. Lane 1, total fraction of cell lysate; lane 2, soluble fraction of cell lysate; lane 3, flow-through; lane 4, wash 1 (10 mM imidazole); lane 5, wash 2 (50 mM imidazole); lane 6, elution 1 (6-fold diluted, 250 mM imidazole); lanes 7 and 8 elution 1; lane 9, elution 2 (250 mM imidazole). The calculated molecular weight of EGFP-Tyr40sTyr was 28.1 kDa.

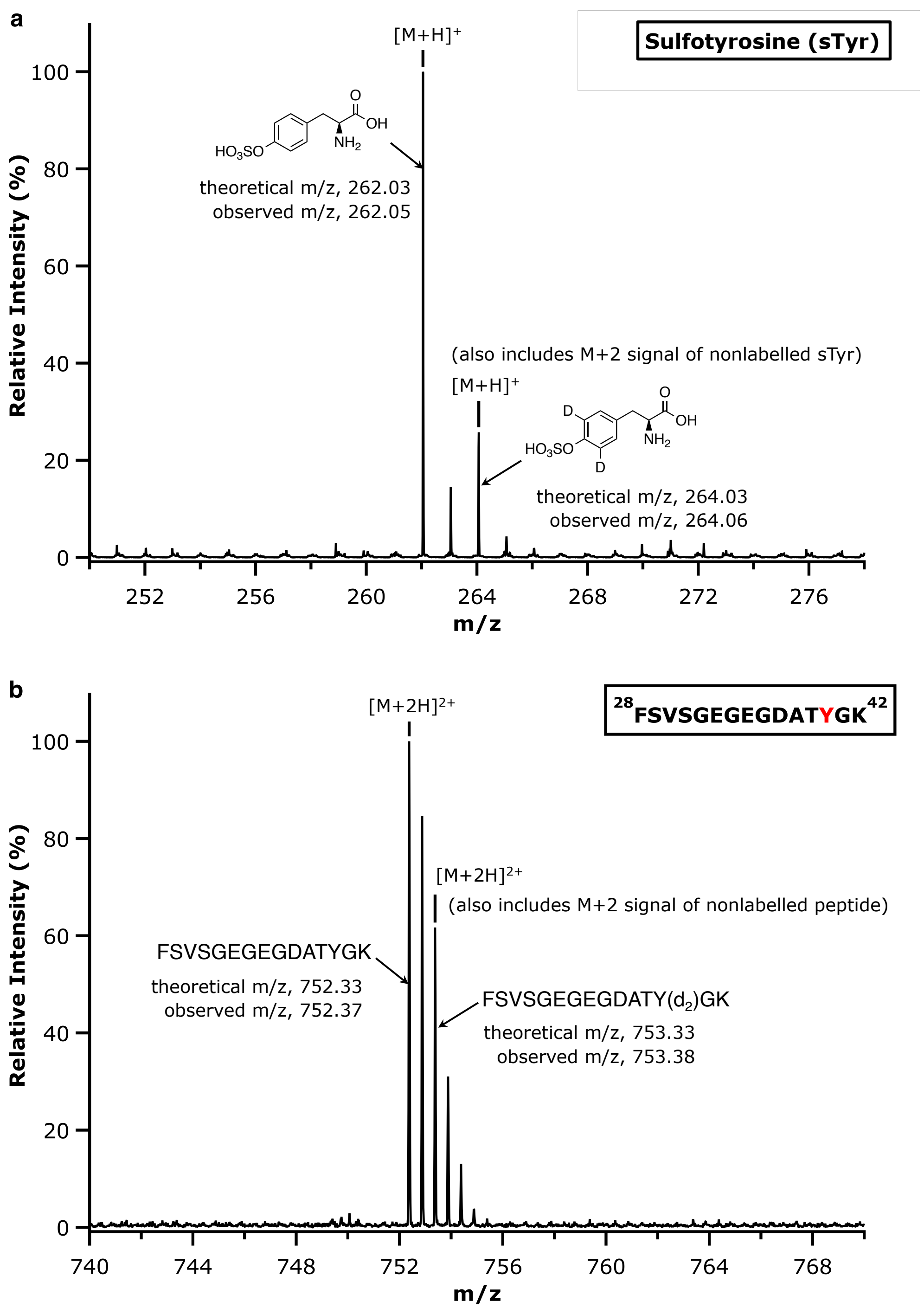

Fig. 5 Mass spectrometry analysis of sulfotyrosine incorporation into protein in mammalian cells. **a** Analysis of sTyr-D_2/sTyr mixture. **b** Analysis of trypsin digested EGFP-Tyr40sTyr that was purified from 293T cells. Calculations of isotope labelling percentage in both small molecule and peptide are described in the Experimental section. Reported m/z values are based on monoisotopic mass.

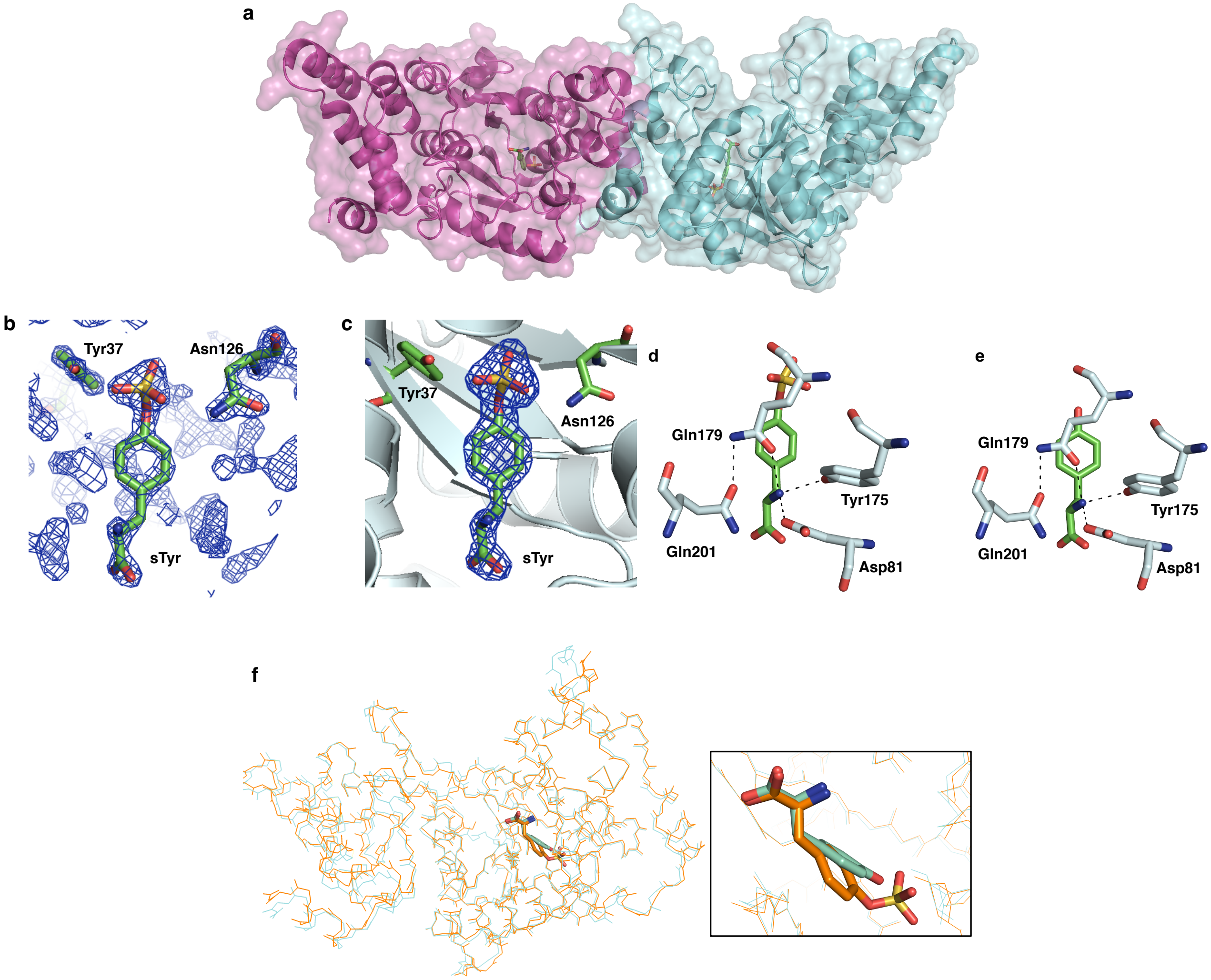

Fig. 6 Structural analysis of sTyrRS. **a** Overall structure of sTyrRS and sTyr complex. The protein ribbon diagrams of the two monomers are shown in purple and cyan, respectively. The sTyr ligands are represented in stick. **b** 2Fo-Fc electron density map (3.0 σ) with focus on sulfotyrosine (sTyr), Tyr37, and Asn126. **c** Fo-Fc omit electron density map (3.0 σ) around the sTyr site of the sTyrRS complexed with sTyr. **d-e** Conserved recognition of the amino group in sTyr (**d**, evolved sTyrRS) and Tyr (**e**, *Ec*TyrRS, PDB ID: 1X8X). Carbon atoms in ligands are shown in green. Carbon atoms in proteins are shown in silver. All oxygen atoms are in red, nitrogen atoms are in blue. Side chains of residue Asp81, Tyr175, and Gln179 form hydrogen bonds with the α-amino group of substrates. **f** Overall structure alignment between the evolved sTyrRS (gold) and wild-type *Ec*TyrRS (PDB ID: 1X8X, cyan). Ligands are in stick representation. A close-up side view of phenol rings of ligands is shown.

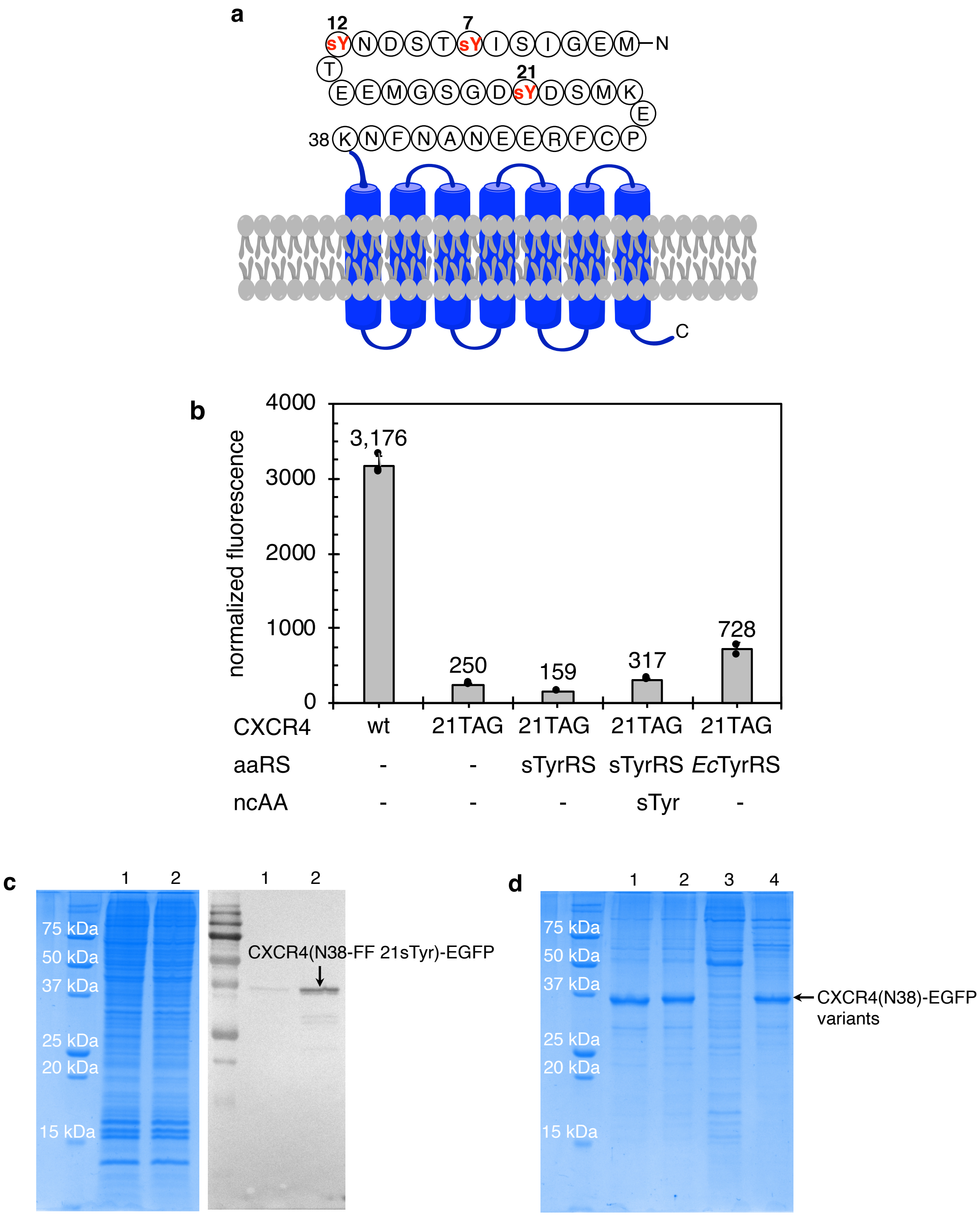

Fig. 7 Site-specific incorporation of sulfotyrosine into CXCR4. **a** Schematics of CXCR4 protein. The 38 amino acid residues and the three sulfation sites on the N-terminus are labeled. **b** Flow cytometry analyses of transfected 293T cells. wt, pCXCR4-EGFP; 21TAG, pCXCR4(21TAG)-EGFP. The normalized fluorescence was calculated by multiplying the mean fluorescence intensity by the

percentage of fluorescent cells in flow cytometry analyses. Each data point is the average of triplet measurements with standard deviation. **c** CXCR4(N38-FF 21TAG)-EGFP expression in 293T cells. Left panel is the image of Coomassie blue-stained SDS-PAGE. Right panel is the image of western blot. Lane 1, no sTyr in medium; lane 2, 1 mM sTyr in medium. (D) SDS-PAGE of protein purified by Ni resin. Lane 1, CXCR4(N38)-EGFP; lane 2, CXCR4(N38-FFF)-EGFP; lane 3, CXCR4(N38-FF 21TAG)-EGFP in the absence of sTyr; lane 4, CXCR4(N38-FF 21TAG)-EGFP in the presence of sTyr (1 mM). **b** Data are plotted as the mean ± standard deviation from n = 3 independent experiments. **b** Source data are provided as a Source data file.

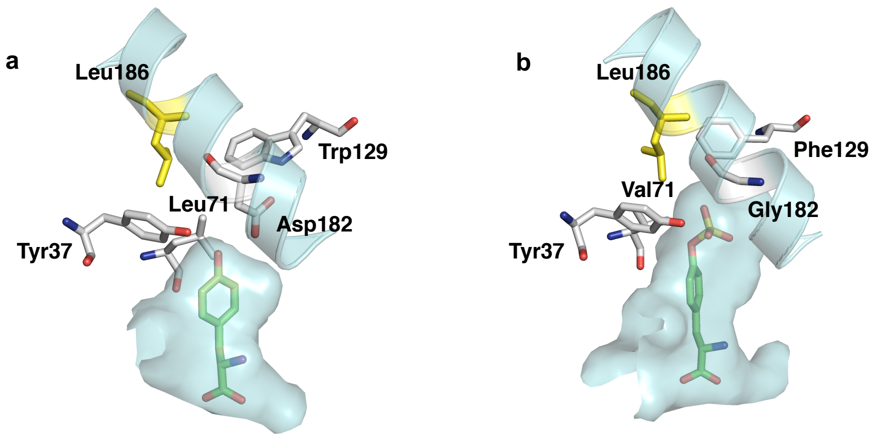

Fig. 8 Active site close-ups. **a** Wild-type *Ec*TyrRS (PDB ID:1X8X). **b** The evolved sTyrRS in this work (PBD ID: 6WN2). The active site surfaces are in cyan. Four residues included in library construction are labelled. Carbon atoms are shown in silver, all oxygen atoms are in red, and nitrogen atoms are in blue. Carbon atoms in ligands are shown in green. An additional residue for consideration, Leu186, is represented in yellow sticks.

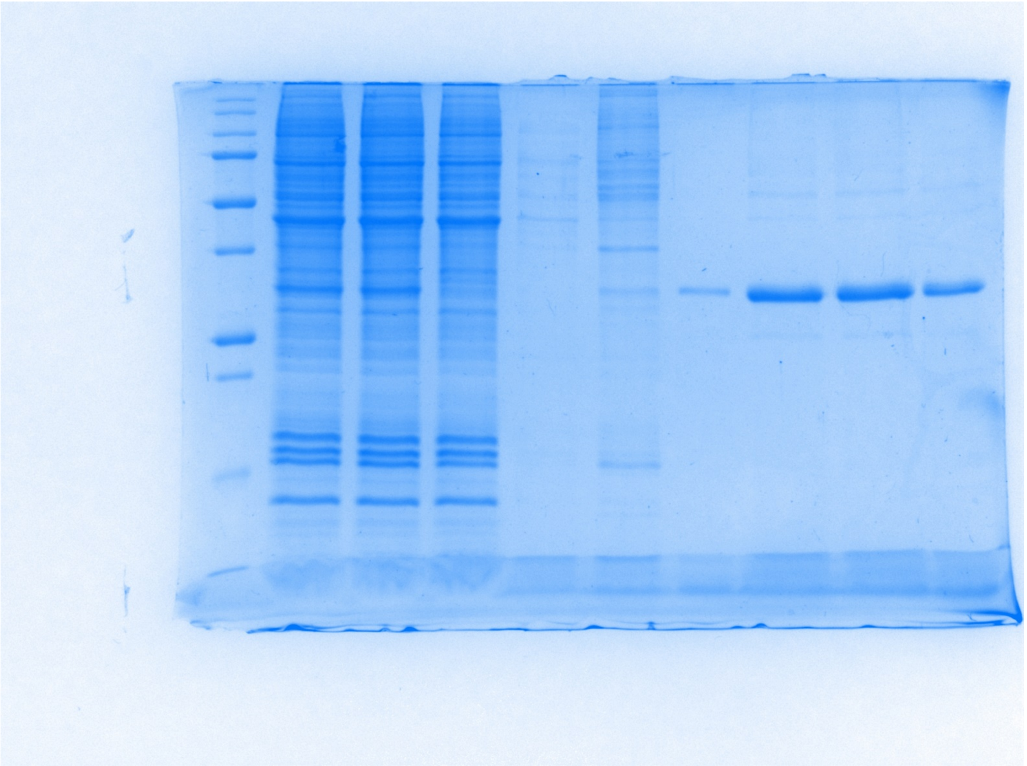

Fig. 9 Original SDS-PAGE of Supplementary Fig. 4b.

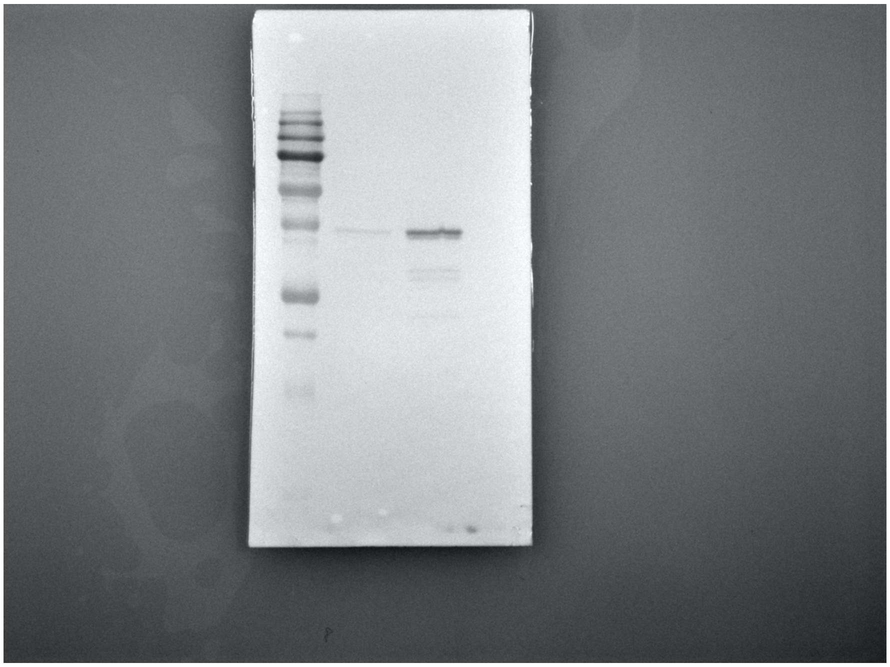

Fig. 10 Original blot of Fig. 4a and Supplementary Fig. 7c.

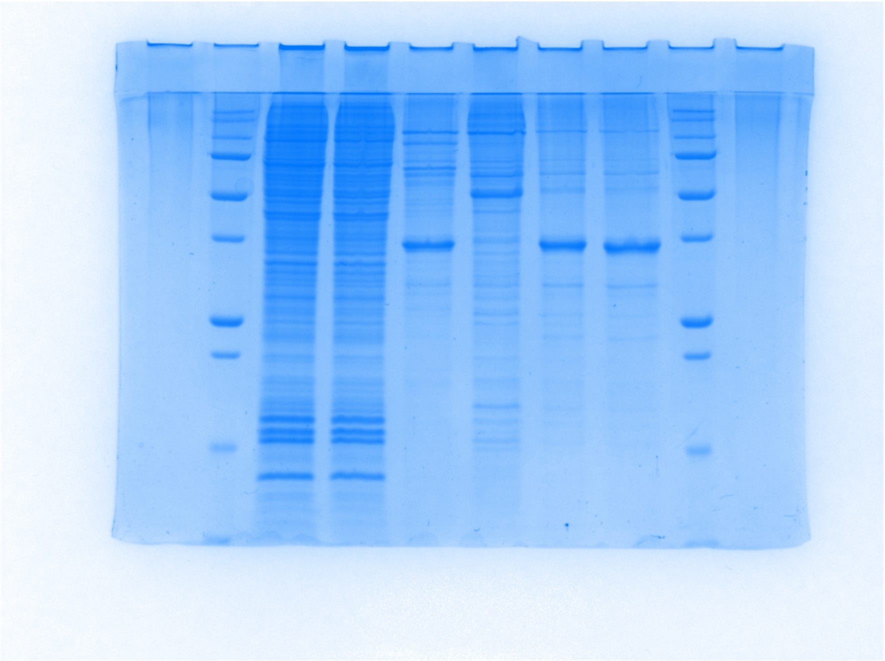

Fig. 11 Original SDS-PAGE of Supplementary Fig. 7c and 7d.

a

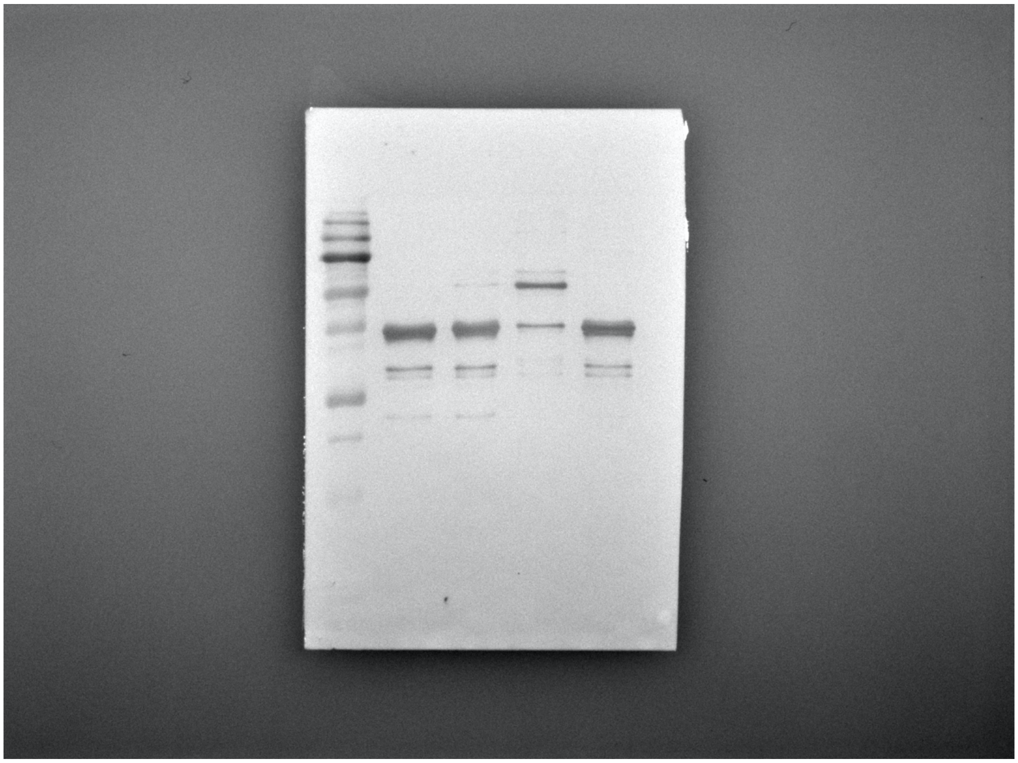

b

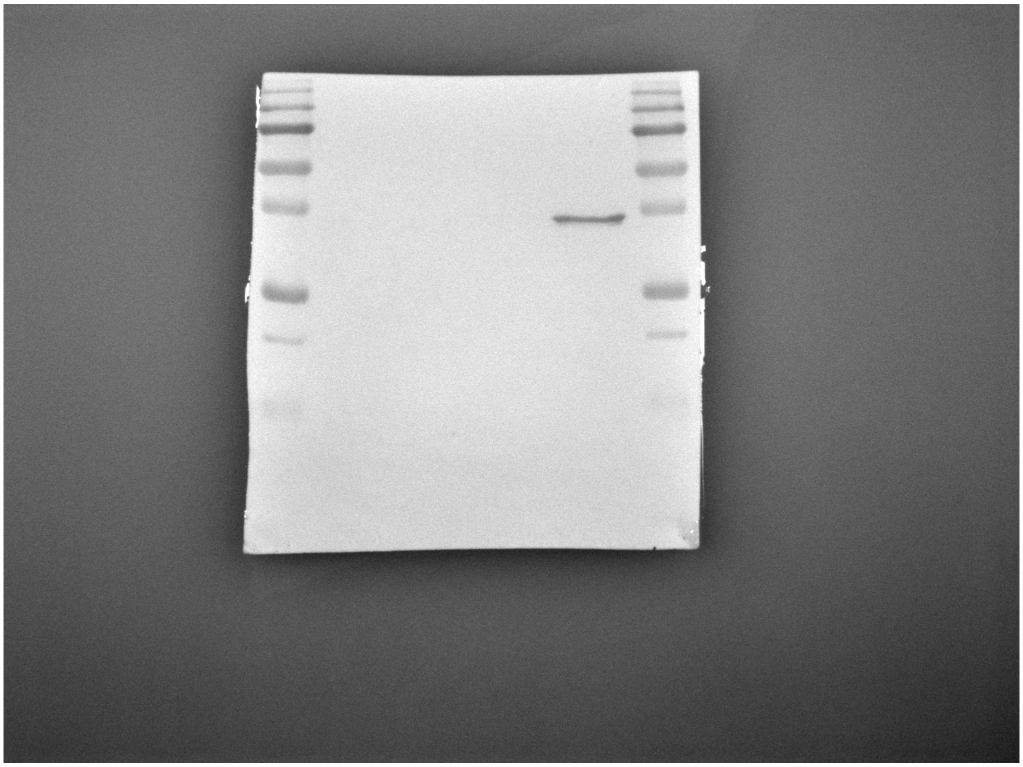

Fig. 12 Original blots of Fig. 4b. **a** Anti-histidine tag antibody. **b** Anti-sTyr antibody.

References

1. Chin, J.W. et al. An expanded eukaryotic genetic code. *Science* **301**, 964-967 (2003).
2. Larson, D.R., Zenklusen, D., Wu, B., Chao, J.A. & Singer, R.H. Real-time observation of transcription initiation and elongation on an endogenous yeast gene. *Science* **332**, 475-478 (2011).
3. Wang, N. et al. Construction of a live-attenuated HIV-1 vaccine through genetic code expansion. *Angew. Chem., Int. Ed.* **53**, 4867-4871 (2014).
4. Yuan, Z. et al. Controlling multicycle replication of live-attenuated HIV-1 using an unnatural genetic switch. *ACS Synth. Biol.* **6**, 721-731 (2017).
5. Heredia, J.D. et al. Mapping Interaction Sites on Human Chemokine Receptors by Deep Mutational Scanning. *J. Immunol.* **200**, 3825-3839 (2018).
6. Ran, F.A. et al. Genome engineering using the CRISPR-Cas9 system. *Nat. Protoc.* **8**, 2281-2308 (2013).
