# Supplementary material for: Functional genetic encoding of sulfotyrosine in mammalian cells
Source: Nat Commun. 2020 Sep 24;11:4820. doi: 10.1038/s41467-020-18629-9 (PMC7515910; doi:10.1038/s41467-020-18629-9)
Supplement: Supplementary file 3 — Reporting Summary [file 41467_2020_18629_MOESM3_ESM.pdf]

nature research

Corresponding author(s): Wei Niu & Jiantao Guo

Last updated by author(s): 8/23/2020

Reporting Summary

Nature Research wishes to improve the reproducibility of the work that we publish. This form provides structure for consistency and transparency in reporting. For further information on Nature Research policies, see Authors & Referees and the Editorial Policy Checklist .

Statistics

For all statistical analyses, confirm that the following items are present in the figure legend, table legend, main text, or Methods section.

n/a	Confirmed	
☐	☒	The exact sample size (n) for each experimental group/condition, given as a discrete number and unit of measurement
☐	☒	A statement on whether measurements were taken from distinct samples or whether the same sample was measured repeatedly
☐	☒	The statistical test(s) used AND whether they are one- or two-sided<br>*Only common tests should be described solely by name; describe more complex techniques in the Methods section.*
☒	☐	A description of all covariates tested
☒	☐	A description of any assumptions or corrections, such as tests of normality and adjustment for multiple comparisons
☐	☒	A full description of the statistical parameters including central tendency (e.g. means) or other basic estimates (e.g. regression coefficient) AND variation (e.g. standard deviation) or associated estimates of uncertainty (e.g. confidence intervals)
☐	☒	For null hypothesis testing, the test statistic (e.g. F, t, r) with confidence intervals, effect sizes, degrees of freedom and P value noted<br>*Give P values as exact values whenever suitable.*
☒	☐	For Bayesian analysis, information on the choice of priors and Markov chain Monte Carlo settings
☒	☐	For hierarchical and complex designs, identification of the appropriate level for tests and full reporting of outcomes
☒	☐	Estimates of effect sizes (e.g. Cohen's d, Pearson's r), indicating how they were calculated

Our web collection on statistics for biologists contains articles on many of the points above.

Software and code

Policy information about availability of computer code

Data collection	BioRad Image Lab (version 5.0), MassLynx 4.1 (Waters Corporation, Manchester UK), FlowJo Collector's Edition 7.5.110.6
Data analysis	MS Excel 365; BioRad Image Lab (version 5.0); MassLynx 4.1 (Waters Corporation, Manchester UK); IGOR Pro 7.0 (Wave Metrics, Lake Oswego, OR, USA); FlowJo Version 10, PyMol (Version 2.3.3), COOT, PHENIX 1.13, REFMAC5, MolProbity 7.

For manuscripts utilizing custom algorithms or software that are central to the research but not yet described in published literature, software must be made available to editors/reviewers. We strongly encourage code deposition in a community repository (e.g. GitHub). See the Nature Research guidelines for submitting code & software for further information.

Data

Policy information about availability of data

All manuscripts must include a data availability statement. This statement should provide the following information, where applicable:

- Accession codes, unique identifiers, or web links for publicly available datasets
- A list of figures that have associated raw data
- A description of any restrictions on data availability

All data generated or analyzed during this study are included in this article and its Supplementary Information. All relevant data are available from the authors upon reasonable request. Source data are provided with this paper. The source data underlying Figs. 1b, 1c, 2b, 4a, 4b, 4c, Supplementary Information Figs. 4, 7 are provided in the Source Data file. Crystal structure of sTyrRS in complex with sTyr is deposited in Protein Data Bank (PDB ID: 6WN2). Crystal structures of E. coli tyrosyl-tRNA synthetase (TyrRS) in complex with L-tyrosine (PDB ID: 1X8X) and E. coli TyrRS (PDB ID: 2YXN) are accessed from Protein Data Bank.

Field-specific reporting

Please select the one below that is the best fit for your research. If you are not sure, read the appropriate sections before making your selection.

☒ Life sciences ☐ Behavioural & social sciences ☐ Ecological, evolutionary & environmental sciences

For a reference copy of the document with all sections, see nature.com/documents/nr-reporting-summary-flat.pdf

Life sciences study design

All studies must disclose on these points even when the disclosure is negative.

Sample size	Reporter expression and enzyme assays were performed in triplicate. Sample size was determined on the basis of a large number of previously reported studies of similar proteins using similar methods.
Data exclusions	No data were excluded from the analyses
Replication	All experiments were reproduced three times. All attempts at replication were successful.
Randomization	None of the experimental methods were required or appropriate for randomization.
Blinding	Investigators were not blinded in this study because no clinical relevant experiments were performed. However, there is no bias for all the data collected in this study.

Reporting for specific materials, systems and methods

We require information from authors about some types of materials, experimental systems and methods used in many studies. Here, indicate whether each material, system or method listed is relevant to your study. If you are not sure if a list item applies to your research, read the appropriate section before selecting a response.

Materials & experimental systems

n/a	Involved in the study	
☐	☒	Antibodies
☐	☒	Eukaryotic cell lines
☒	☐	Palaeontology
☒	☐	Animals and other organisms
☒	☐	Human research participants
☒	☐	Clinical data

Methods

n/a	Involved in the study	
☒	☐	ChIP-seq
☐	☒	Flow cytometry
☒	☐	MRI-based neuroimaging

Antibodies

Antibodies used	mouse anti-6xHis tag monoclonal antibody clone AD1.1.10 (1:3000, MCA1396, Bio-Rad Laboratories), mouse anti-sulfotyrosine antibody clone sulfo-1C-A2 (1:1000, 05-1100, MilliporeSigma), and HRP conjugated goat anti-mouse IgG (1:1000, 1706516, BioRad Laboratories)
Validation	- For clone AD1.1.10, this mouse anti Histidine tag antibody is routinely tested by Bio-Rad in Western blotting on histidine tagged recombinant proteins and reacts against all histidine-tagged proteins so far tested. In Western blotting of bacterial extracts the antibody has been shown not to cross-react with any endogenous products, although some cross-reactivity may be seen with extracts of insect or mammalian cells.<br>- For clone sulfo-1C-A2, this mouse anti-sulfotyrosine antibody is certified by MilliporeSigma for use in Immunoprecipitation, ELISA, and Western Blotting.

Eukaryotic cell lines

Policy information about cell lines

Cell line source(s)	293T cells were purchased from ATCC. CXCR4-knockout 293T cells were constructed in the lab of the corresponding author, Dr. Wei Niu.
Authentication	The commercial 293T cells were not authenticated. The CXCR4-knockout 293T cells were authenticated. by DNA sequencing.
Mycoplasma contamination	All cell lines were regularly mycoplasma-tested and negative.

Commonly misidentified lines
(See ICLAC register)

None

Flow Cytometry

Plots

Confirm that:

- [x] The axis labels state the marker and fluorochrome used (e.g. CD4-FITC).
- [x] The axis scales are clearly visible. Include numbers along axes only for bottom left plot of group (a 'group' is an analysis of identical markers).
- [x] All plots are contour plots with outliers or pseudocolor plots.
- [x] A numerical value for number of cells or percentage (with statistics) is provided.

Methodology

Sample preparation	293T cells (1 x 105) seeded in a single well of a 24 well plate were grown for 24 h, then transfected with one plasmid that encodes a tRNA synthetase (0.8 µg of psTyrRS, AZFRS, or EcTyrRS) and pEGFP (0.8 µg) in 2 µL of Lipofectamine. Transfected cells were cultured for an additional 24 h in 0.5 mL of media without or with ncAA (1 mM of sTyr or AzF). Cells were detached with 0.3 mL of Trypsin/EDTA (0.05%, Thermo Fisher Scientific), washed once with 1 mL DPBS, and collected by centrifugation at 300g for 5 min. Collected cells were resuspended in 0.5 mL 4% paraformaldehyde (w/v) and incubated for 20 min at room temperature. Following removal of the fixation reagent by centrifugation, cells were resuspended in 0.5 mL DPBS and kept on ice until analysis.
Instrument	Cytek DxP10
Software	Data was acquired using FlowJo Collectors Edition , and post data collection analysis was performed using FlowJo Version 10.
Cell population abundance	A total of 30,000 cells were analyzed for each sample. Sorting was not performed for this experiment, it was just analysis of GFP expression.
Gating strategy	The initial "lymphocyte" gate was drawn on the primary population on the FSC/SSC plot. Then those gated cells were drawn on a GFP vs SSC plot. The GFPpositve region was determined based on a non-GFP expressing cell sample. A figure (Supplementary Fig. 2b) exemplifying the gating strategy is provided in the Supplementary Information.

- [x] Tick this box to confirm that a figure exemplifying the gating strategy is provided in the Supplementary Information.
